# Cystic Duct Remnant Leading to Stump Cholelithiasis

**DOI:** 10.7759/cureus.92854

**Published:** 2025-09-21

**Authors:** Ayushi Rathore, Krishnanand Anand, Shivam Nagaich

**Affiliations:** 1 General Surgery, LN Medical College and Research Center, Bhopal, IND

**Keywords:** cystic duct remnant, difficult laparoscopic cholecystectomy, laparoscopic cholecystectomy, mrcp, post-cholecystectomy complication, post-cholecystectomy syndrome, postcholecystectomy syndrome, residual gallbladder, residual gallbladder stump cholelithiasis, stump cholelithiasis

## Abstract

A 42-year-old male presented with a two-month history of right upper quadrant abdominal pain, worsening over four days. Six months earlier, he had undergone a laparoscopic cholecystectomy. Examination revealed no jaundice or tenderness, and liver function tests were normal. The patient’s basic inflammatory markers were within the normal range. Ultrasound revealed a residual gallbladder stump or dilated cystic duct with a calculus, and magnetic resonance cholangiopancreatography (MRCP) demonstrated a 3 × 1 cm stump with an 8 mm hypointense focus. Endoscopic retrograde cholangiopancreatography (ERCP) was unsuccessful due to distorted anatomy. Surgical exploration confirmed a cystic duct remnant containing sludge and concretions, which was excised completely. Postoperative recovery was uneventful, and histopathology revealed chronic inflammatory changes consistent with cystic duct stump pathology.

This case illustrates that residual gallbladder stump cholelithiasis may occur after laparoscopic cholecystectomy. Recognition of this possibility, along with appropriate imaging, enables timely diagnosis. Surgical excision of the cystic duct remnant provides definitive treatment and effective symptom relief in patients with post-cholecystectomy syndrome caused by residual ductal concretions.

## Introduction

Cystic duct remnant stone formation is an uncommon but recognized cause of post-cholecystectomy syndrome (PCS), with an incidence reported between 2.5% and 5% [1]. Although laparoscopic cholecystectomy remains the gold standard for symptomatic gallstone disease, a subset of patients continues to experience biliary symptoms postoperatively [2]. PCS occurs in 10-40% of patients [3] and may present within days or even decades after surgery. It encompasses a wide spectrum of manifestations, including abdominal pain, nausea, vomiting, jaundice, and abdominal distension. Non-biliary causes include peptic ulcer disease, gastroesophageal reflux, irritable bowel syndrome, and chronic pancreatitis, whereas biliary causes include strictures, bile leaks, retained or dropped stones, bilomas, abscesses, long cystic duct remnants, sphincter of Oddi dysfunction, and bile salt-induced gastritis [4]. It is important to distinguish two separate entities: (a) residual gallbladder with retained stones due to incomplete or subtotal excision, and (b) cystic duct stump calculi or sludge (cystic duct remnant syndrome). The latter results from a stump longer than 1 cm left behind, which predisposes to bile stasis and stone formation [5]. Residual stump stones remain a diagnostic challenge, and imaging modalities such as ultrasound, magnetic resonance cholangiopancreatography (MRCP), and endoscopic retrograde cholangiopancreatography (ERCP) are valuable in identifying this rare complication [5]. Awareness of these distinctions is essential for accurate diagnosis and to guide the appropriate surgical approach.

## Case presentation

A 42-year-old male presented to the surgical outpatient department with complaints of right upper quadrant abdominal pain for two months, which had worsened over the previous four days. The pain radiated to the back and was not associated with food intake. The patient denied nausea, vomiting, fever, anorexia, or weight loss.

The patient had undergone laparoscopic cholecystectomy seven months earlier for acute cholecystitis of one month’s duration. Intraoperatively, the gallbladder was grossly distended with dense omental adhesions. Because of the distension and acutely inflamed wall, it was difficult to grasp the gallbladder; therefore, aspiration was performed, yielding approximately 100 mL of purulent fluid. The cystic duct and artery were clipped, and the gallbladder was completely removed.

**Figure 1:**
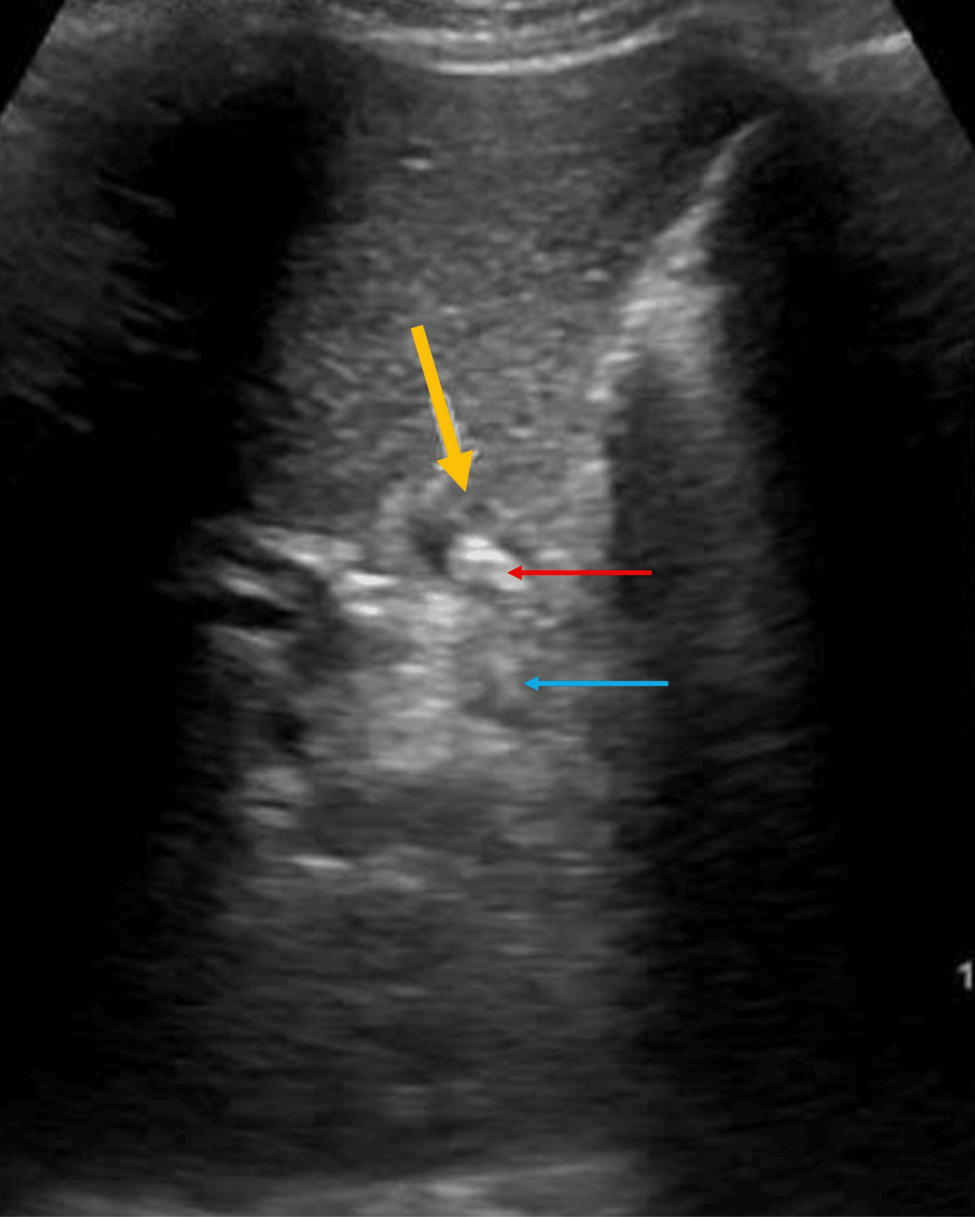
Abdominal ultrasound showing residual gallbladder stump or dilated cystic duct remnant (yellow arrow) with echogenic foci (red arrow) casting posterior acoustic shadow (blue arrow), suggestive of calculus.

On examination, the abdomen was soft and non-tender, and there was no jaundice. Based on this history, cholelithiasis was initially considered unlikely, and the patient was evaluated for alternative causes. Liver function tests, serum amylase, and lipase were all within normal limits, with no biochemical evidence of obstructive jaundice. The patient’s total leukocyte count and C-reactive protein were within normal range, consistent with the absence of systemic infection. Abdominal ultrasound revealed a residual gallbladder stump or dilated cystic duct with a calculus (Figure 1).

**Figure 2:**
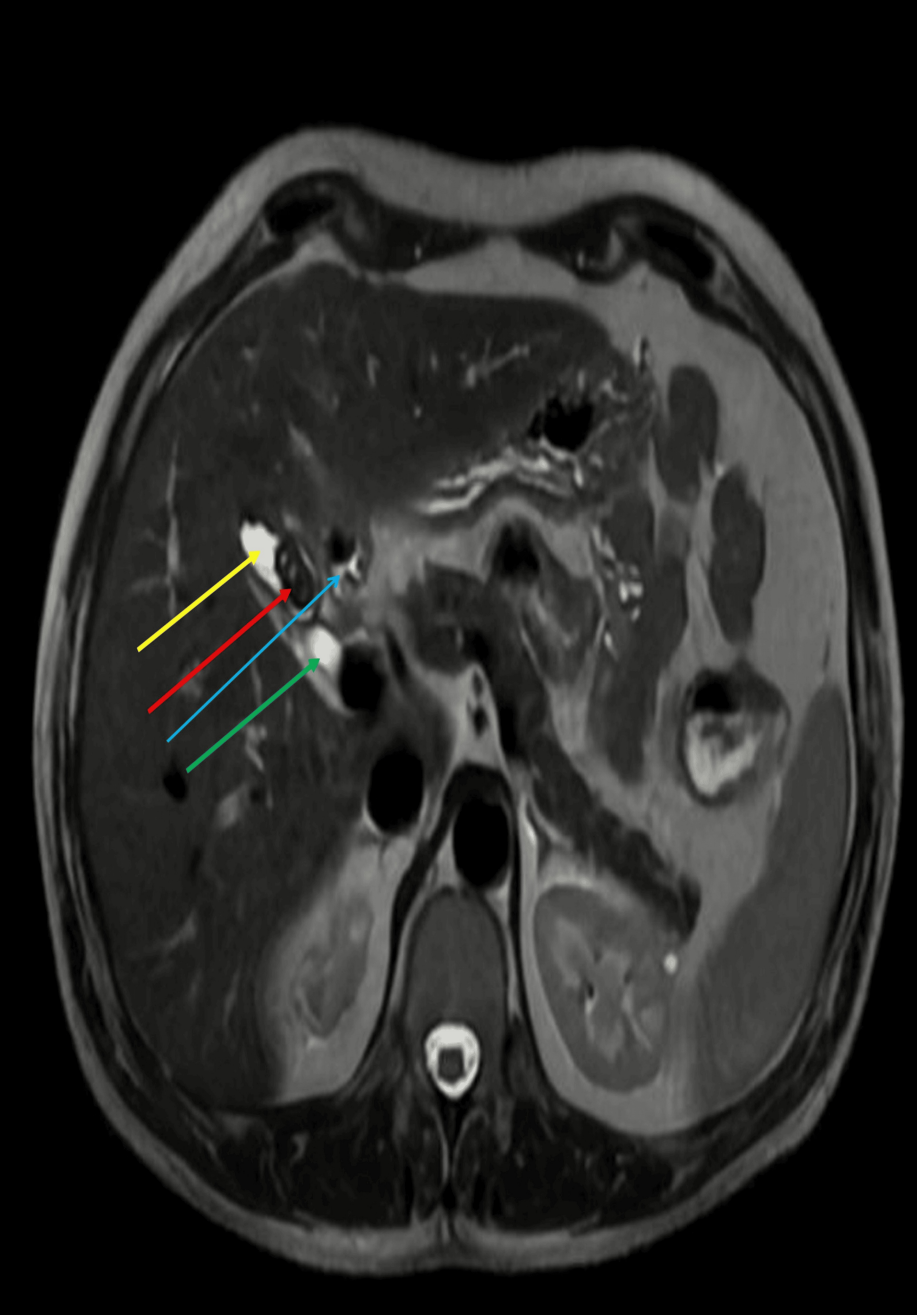
Transverse section of magnetic resonance cholangiopancreatography (MRCP) showing stump of dilated cystic duct (yellow arrow), calculus (red arrow), surgical clip (light blue arrow), and cystic duct (green arrow).

**Figure 3:**
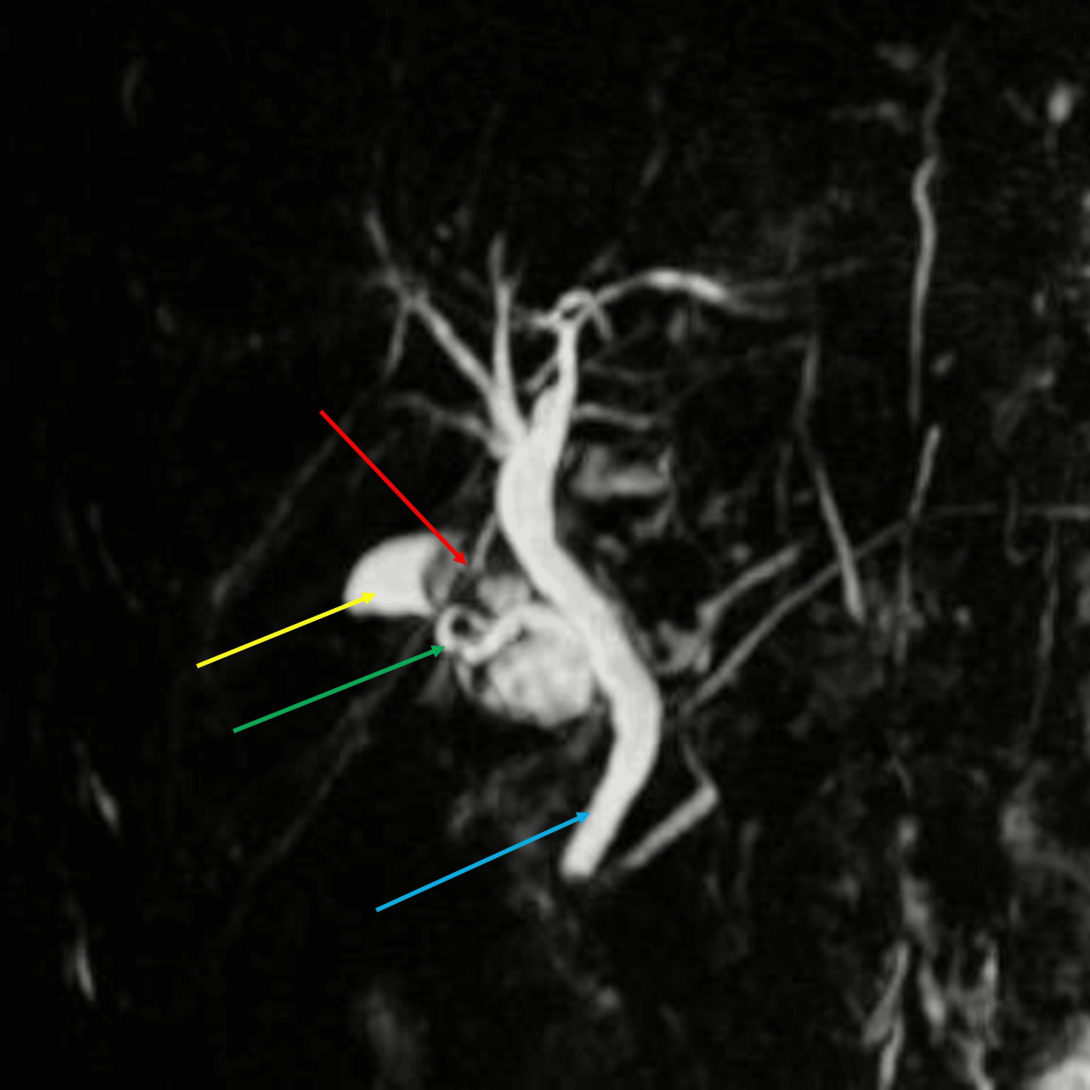
Magnetic resonance cholangiopancreatography (MRCP) showing stump of dilated cystic duct (yellow arrow), calculus (red arrow), cystic duct (green arrow), and common bile duct (blue arrow).

MRCP demonstrated a 3 × 1 cm T2 hyperintense outpouching with an 8 mm T2 hypointense focus in the gallbladder fossa, consistent with a residual gallbladder stump calculus or a dilated cystic duct with a surgical clip (Figures 2, 3).

ERCP was attempted for both diagnostic confirmation and possible therapeutic stone retrieval. However, cannulation was unsuccessful due to the distorted anatomy of the sphincter of Oddi.

**Figure 4:**
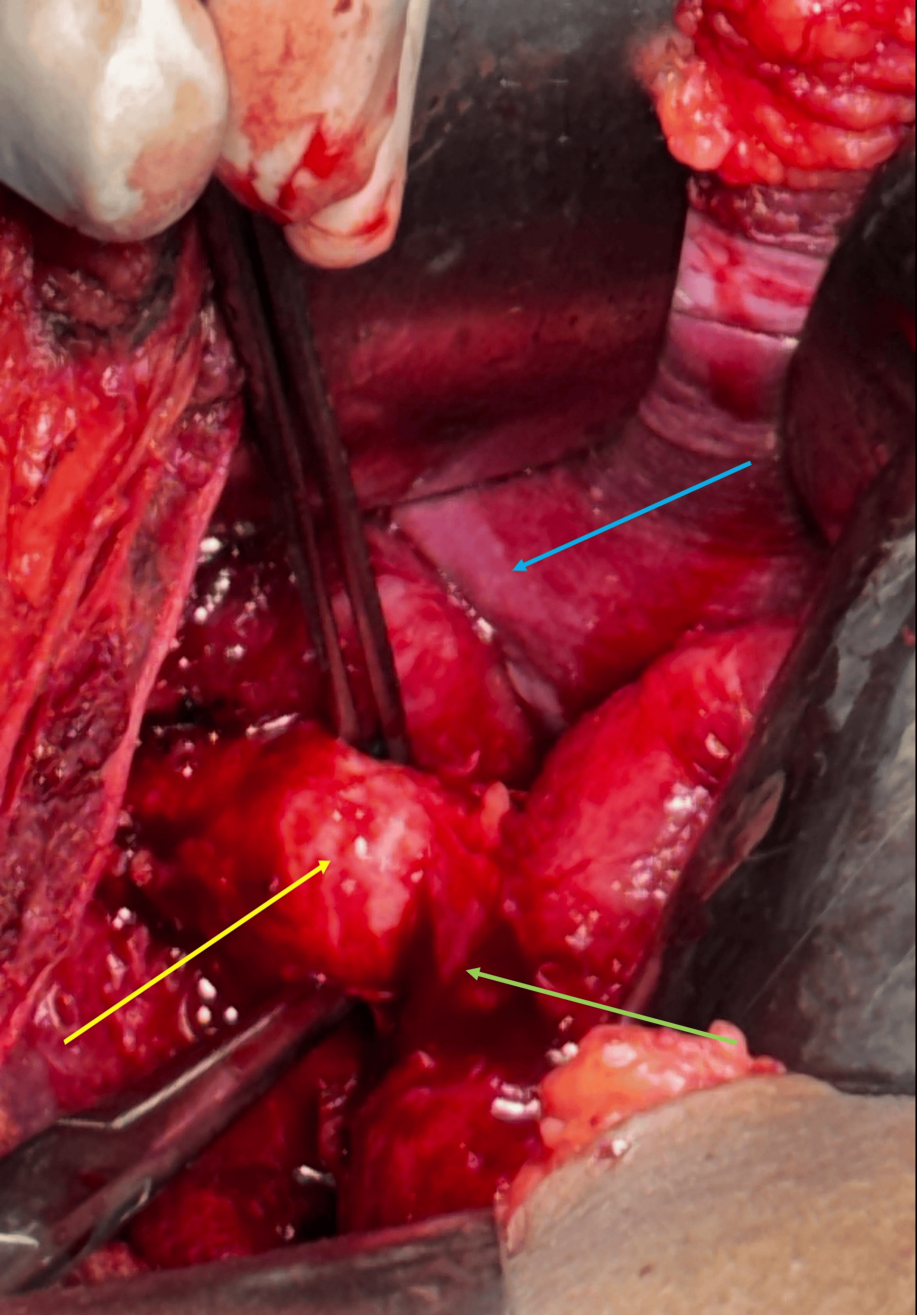
Intraoperative image showing dilated cystic duct remnant (yellow arrow), liver (blue arrow), and bowel (green arrow).

**Figure 5:**
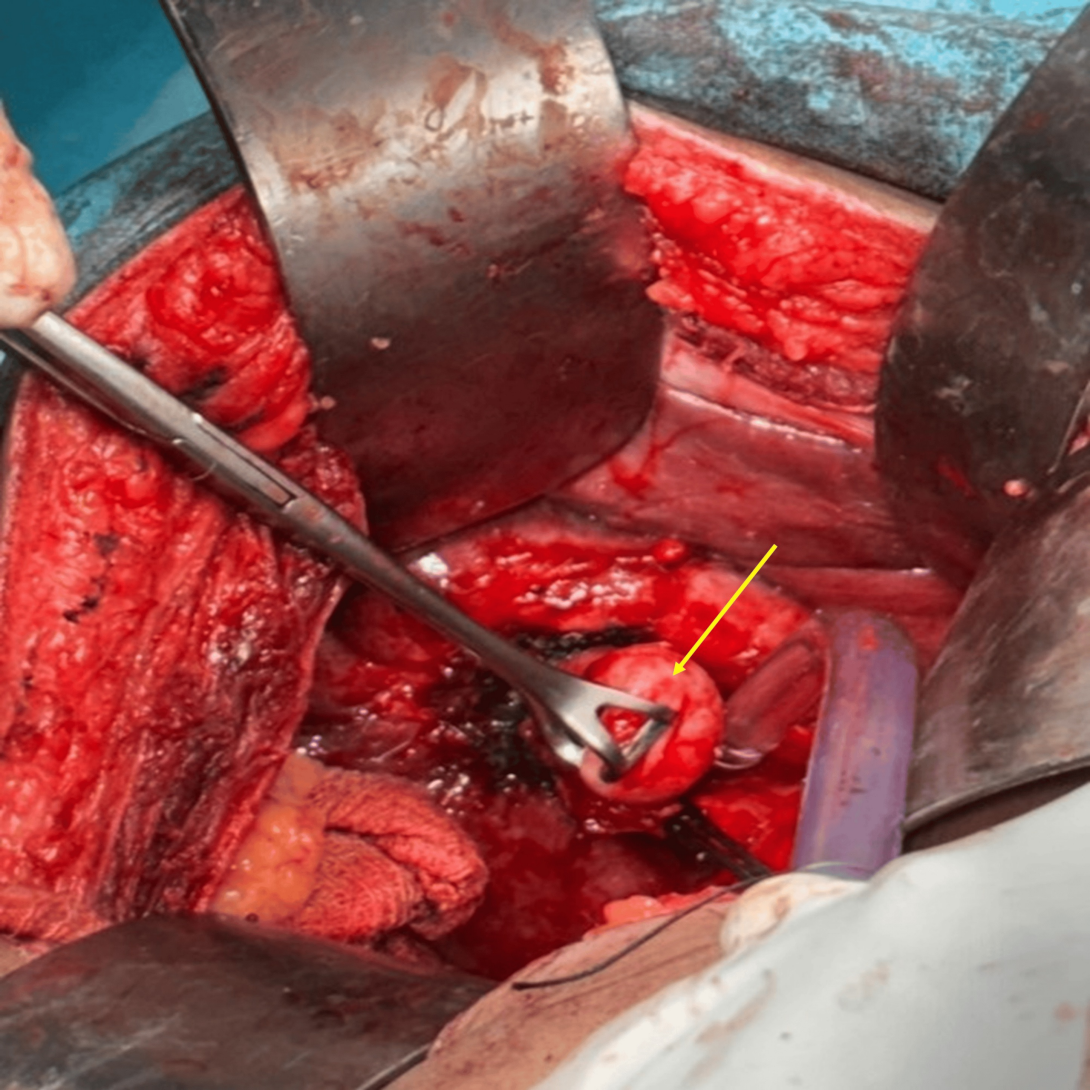
Dilated cystic duct remnant grasped with Babcock forceps (yellow arrow).

**Figure 6:**
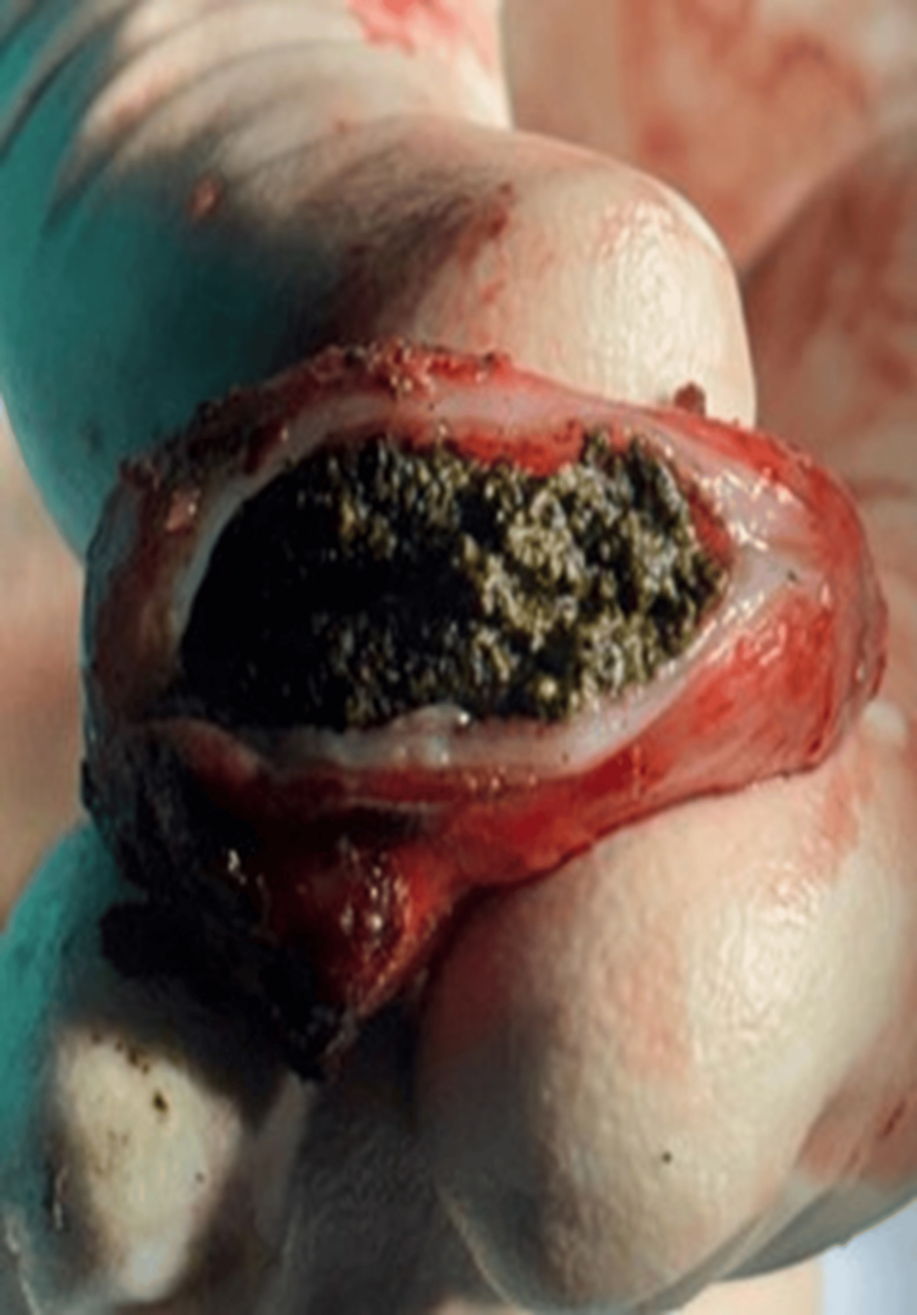
Cut section of the dilated cystic duct remnant showing sludge.

**Figure 7:**
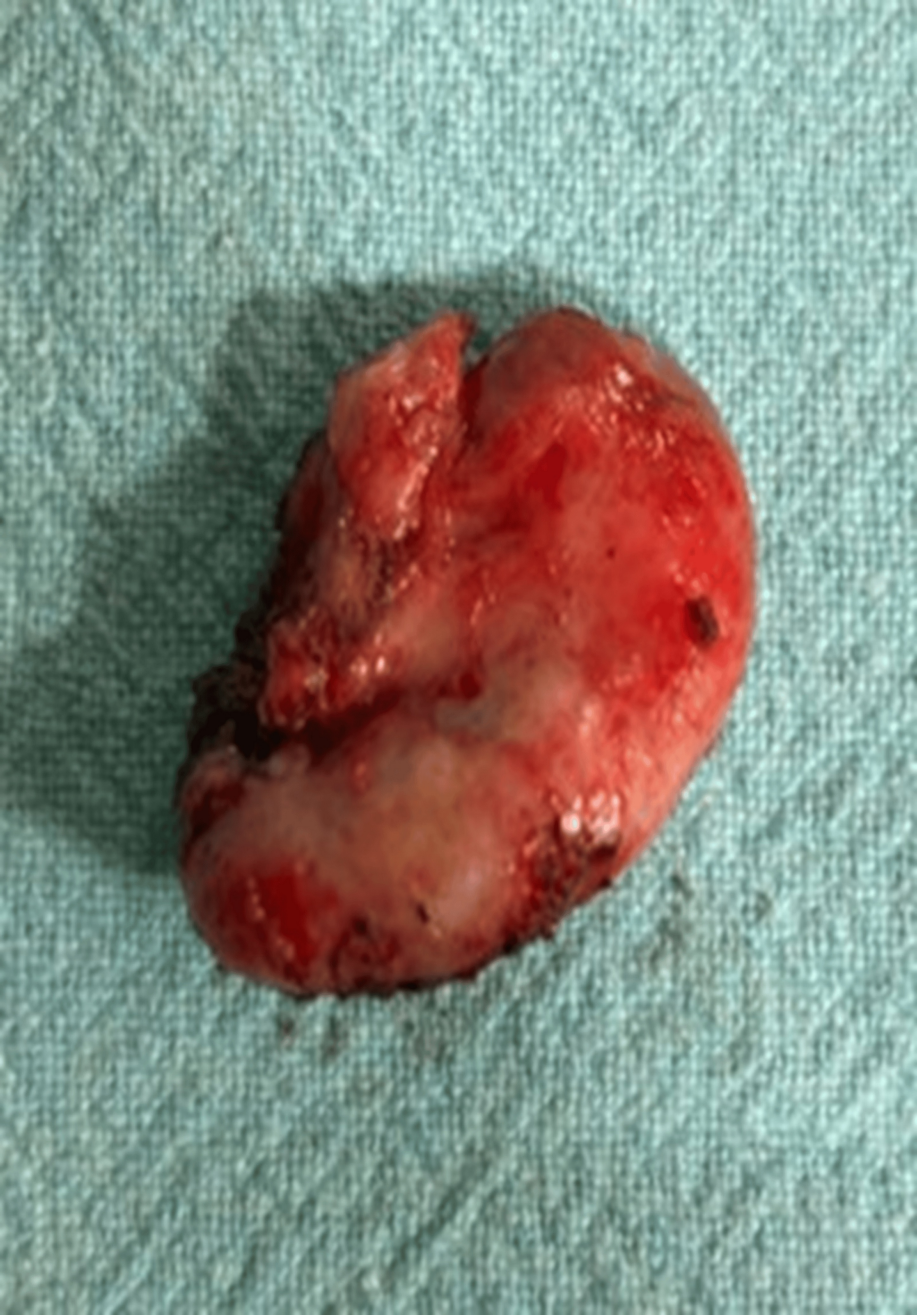
Excised specimen of the dilated cystic duct remnant.

The patient subsequently underwent open surgical exploration through a right subcostal incision. Dense adhesions were encountered and carefully released using sharp and blunt dissection. The common bile duct was identified, and a remnant of the enlarged cystic duct was palpated. The cystic duct remnant was dissected using a right-angled (Mixter) forceps, ligated with suture material, and excised. A size 28 abdominal drain was placed in the gallbladder fossa (Figures 4-7).

The postoperative course was uneventful, and the patient did not require ICU admission. Postoperative management included intravenous antibiotics and analgesics for 48 hours, followed by oral analgesics. The patient tolerated early oral intake, and the abdominal drain was removed on postoperative day three. A summary of the patient’s clinical course, laboratory values, imaging, and interventions is presented in Table 1.

**Table 1:** Summary of clinical course, laboratory findings, imaging, and interventions. LFTs: liver function tests; USG: ultrasound; POD: postoperative day; IV: intravenous; MRCP: magnetic resonance cholangiopancreatography; ERCP: endoscopic retrograde cholangiopancreatography.

Timeline	Laboratory findings	Imaging findings	Intervention/outcome
Admission (Day 0)	Normal LFTs, amylase, lipase; no leukocytosis	Ultrasound: residual gallbladder stump/dilated cystic duct with calculus	Supportive management initiated
Day 1	Stable labs	MRCP: 3 × 1 cm stump with 8 mm focus	–
Day 2	Stable labs	–	ERCP was attempted but was unsuccessful due to distorted anatomy
Day 3 (Surgery, POD 0)	No leukocytosis, stable vitals	–	Open surgical exploration performed; cystic duct stump excised with concretions and sludge. IV antibiotics and analgesics were administered for 48 hours
POD 2	–	–	Oral intake resumed
POD 3	–	–	Drain removed
POD 5	–	–	Patient discharged
Follow-up (6 months)	–	–	Patient remains well and asymptomatic

Histopathological examination revealed chronic inflammatory changes with focal epithelial hyperplasia, consistent with post-cholecystectomy cystic duct stump pathology. The patient was well on a six-month follow-up.

Written informed consent was obtained from the patient for participation in this study and for the publication of this case report, including accompanying images.

## Discussion

PCS refers to the persistence or recurrence of biliary or gastrointestinal symptoms following cholecystectomy. Laparoscopic cholecystectomy provides long-term symptomatic relief in nearly 85% of patients, yet a subset continues to experience symptoms resembling their preoperative state [2,6]. Stump or remnant calculi are particularly challenging to diagnose. Ultrasonography may demonstrate acoustic shadows suggestive of stones, but MRCP and ERCP remain the preferred diagnostic tools, though even these may miss small or anatomically obscured calculi [7-9]. Cystic duct remnant syndrome (CDRS), as in the present case, occurs when a residual cystic duct longer than 1 cm is left behind, with a reported prevalence of less than 2.5% [10]. A long cystic duct stump acts as a blind loop that predisposes to bile stasis, sludge, and subsequent calculus formation [1,10]. Difficult dissections in the setting of empyema, severe inflammation, or dense adhesions often result in a longer residual stump or missed stones, further increasing the risk [1,4,11,12]. Emergency or technically challenging cholecystectomies, including subtotal procedures, are additional contributors to cystic duct remnant pathology [8,9,11,12]. Symptoms may develop soon after surgery or present years later as biliary colic, jaundice, or recurrent infection [7,9]. In our patient, MRCP confirmed biliary PCS. However, ERCP failed due to the distorted anatomy of the sphincter of Oddi. Residual gallbladder with stones requires a complete cholecystectomy, whereas cystic duct stump stones (as in this case) require cystic duct exploration and excision. Although ERCP is typically considered the first-line therapeutic option, recent advances such as laparoscopic re-exploration and laparoscopic cystic duct excision offer safe, minimally invasive alternatives when ERCP fails or is not technically feasible [8,11,12]. In this case, open surgical excision of the stump was performed successfully, providing definitive treatment. Reoperations in the Calot’s triangle can be technically demanding due to chronic inflammation and scarring. Open surgery, however, continues to be a safe and effective alternative, as demonstrated here. This case emphasizes the importance of meticulous surgical technique during the index cholecystectomy to prevent long cystic duct remnant complications. Additionally, thorough imaging and clinical vigilance are critical for the diagnosis of cystic duct stump stones, while complete excision of the stump remains the definitive treatment, offering complete cure.

## Conclusions

Long cystic duct remnants containing sludge or concretions represent a rare but preventable cause of post-cholecystectomy syndrome. In our patient, MRCP confirmed the diagnosis, while ERCP failed due to distorted anatomy. Open surgical excision of the stump was successfully performed and provided durable symptom relief. These findings are consistent with published data demonstrating MRCP’s high diagnostic accuracy, ERCP’s limited therapeutic role due to technical challenges, and the efficacy of surgical excision in achieving symptom resolution. This case underscores the importance of meticulous surgical technique during the index cholecystectomy to avoid leaving a long cystic duct stump. Thorough preoperative imaging, careful intraoperative dissection, and timely reoperation when indicated remain the cornerstones for preventing and managing this uncommon but clinically significant complication.
